# Overexpression of aurora B kinase (AURKB) in primary non-small cell lung carcinoma is frequent, generally driven from one allele, and correlates with the level of genetic instability

**DOI:** 10.1038/sj.bjc.6602779

**Published:** 2005-09-13

**Authors:** S L Smith, N L Bowers, D C Betticher, O Gautschi, D Ratschiller, P R Hoban, R Booton, M F Santibáñez-Koref, J Heighway

**Affiliations:** 1 Gene Function Group, Roy Castle Lung Cancer Programme, University of Liverpool Cancer Research Centre, 200 London Road, Liverpool L3 9TA, UK; 2 Institute of Medical Oncology, University of Bern, 3010 Bern, Switzerland; 3 Human Genomics Research Group, Institute for Science and Technology in Medicine, Keele University School of Medicine, Stoke-on-Trent, UK; 4 Christie Hospital NHS Trust, Manchester, UK; 5 Institute of Human Genetics, International Centre for Life, Central Parkway, Newcastle upon Tyne NE1 3BZ, UK

**Keywords:** lung cancer, aurora, genetic instability, stem cells

## Abstract

Aurora kinases are key regulators of chromosome segregation during mitosis. We have previously shown by microarray analysis of primary lung carcinomas and matched normal tissue that AURKB (22 out of 37) and AURKA (15 out of 37) transcripts are frequently over-represented in these tumours. We now confirm these observations in a second series of 44 carcinomas and also show that aurora B kinase protein levels are raised in the tumours compared to normal tissue. Elevated levels of expression in tumours are not a consequence of high-level amplification of the AURKB gene. Using a coding sequence polymorphism we show that in most cases (seven out of nine) tumour expression is predominantly driven from one AURKB allele. Given the function of aurora B kinase, we examined whether there was an association between expression levels and genetic instability. We defined two groups of high and low AURKB expression. Using a panel of 10 microsatellite markers, we found that the group showing the higher level of expression had a higher frequency of allelic imbalance (*P*=0.0012). Analysis of a number of other genes that are strongly and specifically expressed in tumour over normal lung, including SERPINB5, TERT and PRAME, showed marked allelic expression imbalances in the tumour tissue in the context of balanced or only marginally imbalanced relative allelic copy numbers. Our data support a model of early carcinogenesis wherein defects in the process of inactivation of lung stem-cell associated genes during differentiation, contributes to the development of carcinogenesis.

Lung cancer is the leading cause of cancer-related death (Shibuya *et al*, 2002). Although a small proportion of tumours are sensitive to existing cytotoxic or targeted therapies (Lynch *et al*, 2004), most are resistant to current anticancer treatments. While surgical resection may be curative for a limited number of non-small cell carcinoma (NSCLC) patients, most disease is overtly or covertly metastatic at presentation and consequently, currently incurable. The development of more widely effective targeted agents is therefore necessary not only to improve the treatment of advanced stage nonresectable disease but also to complement future screening capabilities in the areas of secondary and tertiary disease prevention (chemoprevention).

An important step in the rational identification of novel drug targets for such treatments lies in an understanding of the gene expression pattern of the tumour cell and how that pattern relates to other normal cells. We have therefore compared transcript levels in a range of primary lung tumours relative to their matched normal tissue through a comprehensive microarray analysis. This has facilitated the construction of ranked lists of genes, the transcripts of which are frequently and dramatically over-represented in tumours compared to matched normal lung tissue (Heighway *et al*, 2002). As most lung carcinomas arise from a minority component of the tissue – the epithelial lining of the bronchial airways – many tumour over-represented transcripts in our analysis will likely reflect appropriate gene expression in the progenitor cell, wherever that expression is not typical of the differentiated cell majority. The comparison of tumour to normal lung tissue is therefore likely to be an effective way to identify potentially targetable gene products that are not commonly active in the bulk of normal cell types.

Inappropriately upregulated (or gain of function somatically mutated) sequences are likely to make the most effective drug targets. If we further assume that such upregulation is often associated with localised genetic or epigenetic damage, then one approach to identify potentially targetable genes that are pathologically miss-regulated is to compare the allele-specific levels of gene expression in tumour and normal tissues (Ratschiller *et al*, 2003). If there is no imbalance – if transcription is driven in the tumour cell equally from both parental alleles – then such expression is not likely to be the direct result of localised damage (although it could be a direct consequence of damage at a *trans* site). Conversely, where alleles are balanced in genomic DNA, the identification of allelic expression imbalance (AEI) in tumours, but not matched normal tissue (Smith *et al*, 2004), indicates that the increased level of expression for a given candidate may not be a consequence of the normal regulatory processes governing the activity of that gene.

It has been considered likely that lung cancer arises from a stem cell or multipotent component of the bronchial epithelium. Indeed, tumours often appear to share the expression patterns of specific genes with multipotent cells of the airway (Massion *et al*, 2003; Smith *et al*, 2003, 2004). By analogy with other solid tumours (Al-Hajj *et al*, 2003; Singh *et al*, 2003), it is possible that such a stem cell or stem cell-like tumour initiating component is a minority population within the neoplastic lesion. Such an argument is supported by *in vitro* data, for example; only one in 1000–5000 lung cancer cells were found to form colonies in soft agar (Hamburger and Salmon, 1977). Although it might be argued therefore that the analysis of gene expression in the bulk of the tumour mass might be an inefficient way to identify differentially expressed transcripts associated with such a minority component, it nevertheless remains likely that elemental, DNA damage-driven, pathological, stem cell expression patterns will be represented throughout the tumour mass. Indeed, in the context of therapy, it could be argued that the most important new drug targets are likely be constitutively and abnormally activated in both the stem cell progenitors and the aberrantly differentiated progeny. Such inappropriately expressed genes may be detected by microarray or other expression analyses of tumour against normal tissue.

In our earlier genome-wide microarray analysis, we identified the aurora B kinase gene (AURKB) as differentially expressed in a high frequency (>2-fold in 59%, 22 out of 37 cases) of primary NSCLC (Heighway *et al*, 2002). Aurora B kinase, a candidate drug target undergoing preclinical and clinical validation (Keen and Taylor, 2004), is thought to facilitate the correct alignment of chromosomes on the metaphase plate by actively destabilising mono-polar spindle attachments (Dewar *et al*, 2004). Our aim was to investigate whether aurora B kinase deregulation occurs in lung cancer. Specifically, it was our intention to confirm the microarray data suggesting strong tumour cell expression, then to investigate: whether high levels of expression were driven from one or both parental alleles in tumours and normal tissue, whether AURKB was amplified in NSCLC and to determine whether AURKB expression was associated with the level of genetic instability within tumours and patient survival.

## METHODS

### Clinical samples and cell lines

RNA and DNA was extracted from tumour and matched normal lung (NSCLC) tissue samples from 48 Swiss patients (for expression, AEI and genetic instability analysis) and a smaller number (37 patients for AEI analyses) of Liverpool patients (Smith *et al*, 2003, 2004) as part of wider ethically approved studies of gene expression in bronchial tissues – cDNA synthesis from the extracted total RNA was also carried out as described previously (Heighway *et al*, 2002). Normal human bronchial epithelial cells (NHBE5239) were obtained from Clonetics and cultured according to the manufacturer's instructions in BEGM-Bronchial Epithelial Medium (Clonetics). Cells (1 × 10^7^) were split into two flasks and after further growth, were subsequently harvested in either an exponential or confluent, growth arrested state (each flask after different lengths of time in subculture). Total RNA was extracted from these parallel cultures using a standard Trizol protocol and cDNA synthesised as for primary tissue as described in Heighway *et al* (2003).

### Comparative multiplex RT–PCR

Comparative multiplex RT–PCR was used to compare levels of test gene transcripts between normal lung and tumour samples, as described previously (Heighway *et al*, 2002). The test gene transcript (AURKB) was coamplified with a nondifferentially expressed control transcript (APPBP2 – KIAA0228) in 30 cycle RT–PCR reactions. Products were run on 3% agarose gels containing 0.5 *μ*g ml^−1^ ethidium bromide, in 0.5 × TBE and visualised by UV illumination. Relative expression was quantified on an Agilent 2100 Bioanalyser using DNA 1000 LabChips® (Agilent Biotechnologies). The peak height of the test gene was divided by that of the control gene within each sample. The comparative value in the patient tumour tissue was then divided by that from the matched normal to calculate the fold difference in expression of the test gene in each patient. For the purpose of this analysis, differential expression of the test gene of ⩾2-fold in the tumour was arbitrarily considered to indicate a relative over-representation of that gene in the tumour. Primers used for APPBP2 were 5′ gaactgtgtgcactcctatttg and 5′ ccgtgccaaatacactgcatgt and for AURKB, 5′ cagagagatcgaaatccaggc and 5′ ccttgagccctaagagcagat. Primers were confirmed to be cDNA specific by test amplifications of genomic DNA.

### Allelic imbalance (AI) and AEI analysis

Allelic (im)balance relates to the genomic balance of the two parental alleles in a DNA samples; allelic expression (im)balance refers to the relative levels of expression of each of those alleles in a cDNA sample. A tissue may therefore show allelic balance but AEI if one parental allele is expressed more strongly than the other. Measurements of AI and AEI were carried out using single-nucleotide polymorphisms (SNPs) localised with the mRNA coding region of each specific gene (cSNPs) in simple PCR (AI) and RT–PCR (AEI) restriction fragment length polymorphism (RFLP) assays. Single-nucleotide polymorphisms were selected from public domain databases and validated in PCR-RFLP assays of DNA extracted from normal lung tissue. Exon-spanning primers were designed around the SNP and confirmed to be cDNA-specific in test amplifications. Subsequently, the patient population (normal DNA) was scanned to identify informative (heterozygote) individuals. Allelic balance in normal and tumour DNA, and in normal (where a product was amplifiable) and tumour cDNA was measured for each informative patient.

#### AURKB

A nonsynonymous C/T (Thr/Met) polymorphism in exon 9 (nt 950 in NM_004217) creates a natural *Nco*I RFLP, with the T (CCATGG) but not the C (CCACGG) allele cleaved by the restriction enzyme. DNA specific (5′ cagaatgggtagtcaaggaag and 5′ gcgacagattgaagggcagag) or cDNA specific (5′ caacgagacctatcgccgc and 5′ gcaccctccgagagttggccc) primer pairs were used in 25 *μ*l PCR (or RT–PCR) reactions containing 1 *μ*l (0.1 *μ*g) of genomic DNA (or 1 *μ*l of cDNA), 0.25 *μ*l of each primer (0.5 *μ*g *μ*l^−1^) and 12.5 *μ*l of Qiagen HotStarTaq Master Mix and 11 *μ*l of dH_2_O. Reactions were cycled at 95°C for 15 min, 30 (RT–PCR, 35) cycles of (94°C for 30 s, 58°C for 90 s, 72°C for 90 s) followed by 30 min at 60°C in a Biometra T3 Thermocycler. Products were visualised on 2.5% agarose gels.

*RFLP-PCR and RFLP-RT–PCR analyses*: A 3 *μ*l aliquot of each PCR product (∼0.5 *μ*g) was digested with 3 U of restriction endonuclease in 20 *μ*l reactions at 37°C for 5 h. Digests were visualised by gel electrophoresis and relative allelic expression quantified by running 1 *μ*l aliquots of the digest on an Agilent 2100 bioanalyser (Agilent Technologies) across a DNA 1000 LabChip®. Ratios of the peak height of the smaller cut band (corresponding to the T allele) to the larger uncut band (corresponding to the C allele) for each of the heterozygous genomic DNA and cDNA samples were calculated. The mean and the standard deviation of the values for the normal genomic DNA samples and the normal cDNA samples were determined, and tumour genomic DNA and the tumour cDNA samples that deviated by more than three standard deviations (s.d.) from the mean of the corresponding normal samples were scored as showing AI or AEI.

#### SERPINB5

A nonsynonymous A/G (Iso/Val) polymorphism in exon 7 (nt 1022 in NM_002639) creates a natural *Hpy*8I RFLP with the G (GTCCAC) but not the A (ATCCAC) allele cleaved by the restriction enzyme. DNA (5′ gtggactaatcccagcaccat and 5′ ctatggaatccccaccatcttc) and cDNA specific 5′ cagtgcagatgatgaacatgg and 5′ ctatggaatccccaccatcttc primers were used in PCR/RT–PCR reactions with the conditions described for AURKB. Reaction products were digested as described but using only 0.5 U of enzyme/reaction for 5 h. Allelic ratios were determined as for AURKB but as so few normal lung cDNAs gave an analyzable product, both AI and AEI were scored with reference to the mean and s.d. values of normal DNA digests.

#### PRAME

A nonsynonymous A/G (Trp/Arg) polymorphism in exon 3 (nt 305 of NM_206956) generates a natural *FauI* RFLP with the G (CCCGC) but not the A (CCCAC) allele cleaved by the restriction enzyme. DNA (5′ caggaggccgttgcttcgta and 5′ cgtctactgtgagggacctc) and cDNA specific (5′ cagtgcagatgatgaacatgg and 5′ ctgccagctccacaagtctc) primers were used in PCR/RT–PCR reactions with the conditions described and digests carried out using 0.6 enzyme units/reaction at 55°C for 5 h. AI and AEI were scored as for SERPINB5.

#### TERT

A synonymous (A/G) polymorphism in exon 2 (nucleotide 970 of AF015950) creates a natural *Psp*0M1 RFLP with the G (GGGCCC) but not the A (AGGCCC) allele cleaved by the restriction enzyme. DNA primers (5′ cgaagaagccacctctttggag and 5′ cagggagatgaacttcttggtgt) were used in standard PCR reactions and products digested with 3 enzyme units/reaction at 37°C for 3 h. Tumour AI was scored by visual comparison with normal DNA products. Given the apparently low levels of telomerase mRNA in primary tumour tissue, a nested protocol was used for RT–PCR. Initial products (38 cycles with 5′ cgaagaagccacctctttggag and 5′ tacacactcatcagccagtgca) were diluted 1/50 and a 1 *μ*l aliquot reamplified with internal primers (5′ tttggagggtgcgctctctg 5′ caggatctcctcacgcagac). Products were digested as for DNA. Only one allelic band was visible in tumour RT–PCR products and so relative quantitation was not attempted.

### Western analysis

Western analysis of protein extracts of tumour and normal lung tissue were carried out essentially as described (Smith *et al*, 2003). Equal amounts of protein from matched tumour and normal tissue were loaded per lane. Protein was detected using mouse monoclonal anti-Aim-1 antibody (1 : 500 dilution; BD Transduction Laboratories) and goat anti-mouse HRP-conjugated secondary antibody (1 : 2000; Dako). Detection was performed using the ECL Plus Western Blotting Detection System (Amersham).

### Quantitative real-time RT–PCR

Gene expression relative to the nondifferentially expressed control gene, APPBP2 was measured using Applied Biosystems (AB) predesigned TaqMan cDNA-specific assays: for APPBP2 (Hs00197271_m1), AURKB (Hs00177782_m1), AURKA (Hs00269212_m1) and SERPINB5 (Hs00184728_m1). It should be noted that the real time assays measured expression at a different point within the cDNA to the comparative multiplex assays. Triplicate 20 *μ*l RT–PCR reactions for each sample containing: 10 *μ*l of AB TaqMan Universal PCR Master Mix, 1 *μ*l of the relevant 20 × assay, 1 *μ*l of target cDNA and 8 *μ*l of dH_2_O were cycled at 50°C for 2 min, 95°C for 10 min and 45 times at 95°C for 15 s, 60°C for 1 min on an AB 7500 Real Time PCR System. *C*_T_ values were calculated using the 7500 SDS software. AURKA and AURKB gene expression for each sample was normalised to control gene expression and fold difference between tumour and normal tissue were calculated using the ΔΔ*C*_T_ method (Applied Biosystems User Bulletin #2, 1997).

### Quantitative real time PCR

Relative gene copy number was quantified using the ΔΔ*C*_T_ of test and control genes in amplifications of normal and tumour tissue derived DNA. Control loci which in our experience do undergo gene amplification or homozygous deletion in primary lung carcinomas were selected. For the initial analysis, the MGMT locus was used (10q26.3 – defined by NM_002412). Taqman PCR assays were designed for this locus, a second control region (gamma globin – HBG, 11p15.4, locus defined by X55656) and for the test gene: AURKB, using Applied Biosystems Primer Express v2.0 software and standard parameters. Triplicate PCR reactions for each sample containing 20 *μ*l of AB TaqMan Universal PCR Master Mix, ∼200ng of genomic DNA, PCR primers (1 mM) and a TaqMan probe (250 nm) in a total of 40 *μ*l were cycled under the standard AB conditions detailed above. The Δ*C*_T_ between the AURKB and MGMT amplifications were calculated for each tumour and compared to a mean Δ*C*_T_ derived from 20 normal lung DNA samples. Tumour samples with Δ*C*_T_ values greater than the normal mean±3 standard deviations were considered to show significant shifts in relative gene copy number from normal DNA. Subsequently, for a small number of samples a ΔΔ*C*_T_ value was calculated by comparing tumour DNA to its matched normal tissue derived DNA. Primer sequences used were, for MGMT, 5′ gcagggtaacgcatagccttac and 5′ tcgcagcacactgttttgaga with a TaqMan probe of 5′ 6-FAM cacaaccgcaaccacgagtccgt TAMRA 3′; AURKB, 5′ tccctgttcgcattcaacct and 5′ gtcccactgctattctccatcac with a TaqMan probe of 5′ 6-FAM ctgttctctccttagctgcccctggc TAMRA 3′; HBG 5′ gcccttgtggatgcctaaga and 5′ gtctctcccttgaaatgctgtga with a TaqMan probe of 5′ 6-FAM cccatgccctcaagtgtgcagattg TAMRA.

### Multiplex microsatellite analysis

Proprietary PCR primers for the microsatellite loci D3S1263, D3S1300, D3S1566, D5S644, D9S157, D9S161, D13S153, D13S171, D13S263, D17S938 were selected from Applied Biosystems linkage sets. These markers have previously been shown to show consistent allelic imbalance in lung carcinoma samples (Liloglou *et al*, 2001). For each locus the forward primer had 5′ fluorescent modification and the reverse primer had a 5′ biotin modification Multiplex PCR reactions (10 *μ*l) containing 2 *μ*l (0.2 *μ*g) of genomic DNA were cycled on a Biometra T3 Thermocycler. In addition to target DNA, each reaction contained following primers: D3S1263 (2.5 pmol), D3S1300 (2 pmol), D3S1566 (1 pmol), D5S644 (2.2 pmol), D9S157 (5 pmol), D9S161 (1.7 pmol), D13S153 (2 pmol), D13S171 (2 pmol), D13S263 (2 pmol), D17S938 (4 pmol); 5 *μ*l of Qiagen Multiplex PCR Kit Master Mix and 2 *μ*l dH_2_O. Cycling conditions were 95°C for 15 min, 30 cycles (94°C for 30 s, 56°C for 90 s, 72°C for 60 s) followed by 30 min at 60°C.

PCR products were purified using 2 *μ*l Dynabeads® M-280 Streptavidin and resuspended in 4 *μ*l of loading buffer (10 : 2 : 1 formamide, dextran blue/EDTA, ROX 400HD size standard). Samples were denatured at 95°C for 5 min, chilled on ice and 1 *μ*l was loaded on a 5% denaturing polyacrylamide gel on a 377 ABI sequencer. The gel image was analysed using the ABI Genescan™ and Genotyper™ software. Allelic imbalance was scored according to previously established performance criteria if the allelic ratio (A1/A2 tumour)/(A1/A2 normal) was outside a range of 1.25–0.75 (Liloglou *et al*, 2000). Assays were carried out in duplicate and AI scored only if both results for an individual sample were outside the normal range.

### Statistical analyses

Comparisons were carried out using a 2 × 2 contingency table running two-tailed Fisher's exact tests (GraphPad). For associations with survival, patients were further placed into two groups according to their AURKB expression levels (real-time). Survival differences were analysed using Kaplan–Meier survivor function estimators and log-rank test (StatView software version 5.0.1 SAS Institute Inc., Cary).

## RESULTS

### AURKB expression in tumours and patient survival

**Figure 1:**
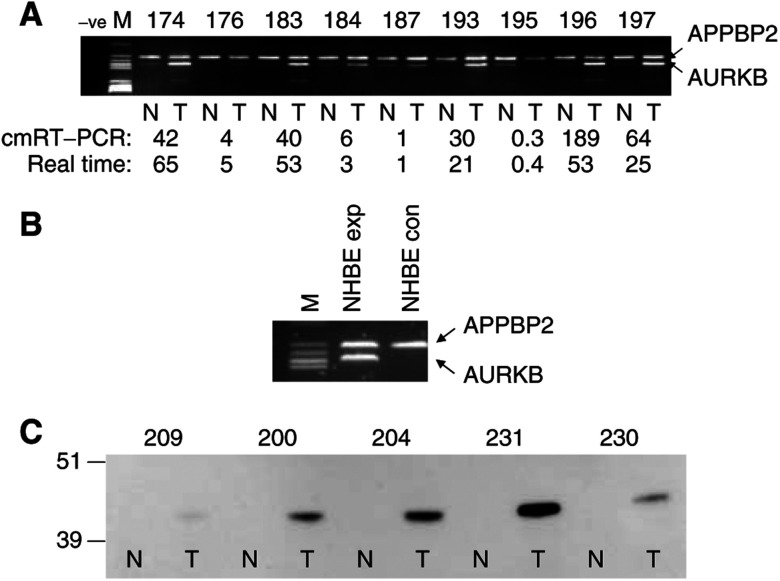
Representative examples of comparative multiplex RT–PCR analyses of matched normal and tumour tissue (**A**) and normal human bronchial cells (**B**) are shown. (**C**) A Western analysis confirming strong differential expression of aurora B kinase between tumour and matched normal tissue in five tumours. Although approximately equal amounts of protein were loaded, absolute aurora B kinase levels, while clearly higher in tumour in each case, do not appear in all cases to show a close relationship to relative transcript levels.

In addition to the noted over-representation of AURKB transcripts in NSCLC, further analysis of the original gene expression array data set (Heighway *et al*, 2002) indicated that AURKA transcripts were also over-represented, but to a lesser extent, in 15 out of 37 cases. AURKC was not scored as over-represented in any tumour (0 out of 37) (data not shown). Using comparative multiplex RT–PCR (cmRT–PCR) in a second series of patients, we were able to confirm that the observed differential expression events were in fact common in primary lung carcinomas (Figure 1). Expression of AURKB was detected by RT–PCR in 44 NSCLC and matched normal lung samples (four Swiss samples gave no usable cDNA following attempted RT–PCR). Using a nondifferentially expressed control gene (APPBP2), cmRT–PCR was used to quantify the degree of relative AURKB expression between the normal and tumour tissues for each patient (Figure 1, Table 1). Using an arbitrary threshold of two-fold or above, 93% (41 out of 44) tumours showed over-representation of this transcript relative to normal lung. Elevated levels of aurora B kinase protein were confirmed in representative matched tumour and normal tissue samples by Western analysis (Figure 1).

**Figure 2:**
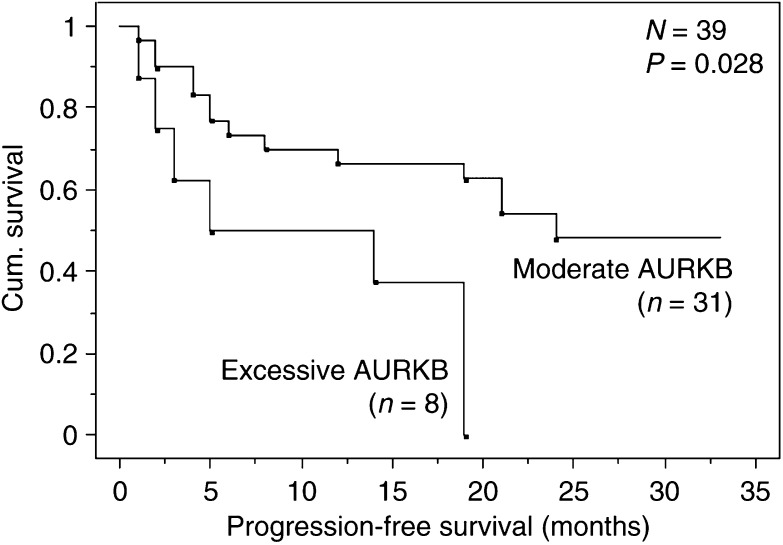
Progression-free survival of the 39 NSCLC patients with AURKB overexpression (relative expression ⩾2) according to the real-time PCR data presented in Table 1. A threshold of 45.8, representing the mean expression value of these patients, was used to separate patients into two groups, representing excessive (*n*=8) and moderate (*n*=31) AURKB overexpression. The Kaplan–Meier method and log-rank test was used.

In a confirmation of the cmRT–PCR data, Taqman real-time ΔΔ*C*_T_ quantitative RT–PCR (qRT–PCR) was used to examine the expression of AURKB relative to APPBP2 in the same 44 samples (Table 1). This analysis again confirmed that AURKB was commonly strongly expressed in the majority of primary NSCLC tissues compared to matched normal tissue (39 out of 44; 89%, showed a two-fold or greater overexpression). Although real-time qRT–PCR is more sensitive than cmRT–PCR, and can therefore measure differential expression in paired samples where expression is at very low absolute levels, there was a good general agreement between the two approaches (Table 1). Survival analysis of the 39 out of 44 patients with AURKB overexpression (relative expression ⩾2 according to the real-time PCR data) were preformed using Kaplan–Meier method and log-rank test (Table 1). A threshold of 45.8 was defined, representing the mean AURKB expression value, dividing the patients into a group with excessive AURKB overexpression and moderate AURKB overexpression. Progression-free survival was significantly shorter for patients with excessive AURKB overexpression (*P*=0.028) (Figure 2), whereas overall survival was not statistically different (*P*=0.153) (data not shown).

In order to determine whether the elevated expression of aurora B kinase was a normal characteristic of proliferating bronchial cells, we firstly examined AURKB expression levels in a primary cell culture model under different conditions. The early-passage primary culture was split into two flasks and the cells were allowed to grow for different lengths of time. From the first flask, RNA was isolated when the population was subconfluent and the cells in exponential growth. In the second parallel flask, the cells were grown to confluence and held for 2 days in this state before RNA was isolated. Using comparative multiplex (Figure 1) and real time qRT–PCR, we were able to demonstrate that AURKB was expressed strongly in proliferating normal primary bronchial epithelial cells, but that this expression was strongly downregulated in quiescent cells (∼4000-fold; ΔΔ*C*_T_ of 12 cycles). AURKA expression was also reduced in confluent relative to exponentially growing cells but to a lesser extent (∼50-fold, ΔΔ*C*_T_ of 5.6 cycles). In each case APPBP2 was used as the qRT–PCR control gene. This raised the possibility that the strong expression of AURKB seen in tumours was merely a consequence of the higher proliferative state of tumour over normal tissue. If this were the case, then it might be expected that both parental alleles would be expressed equally in tumour tissue. We therefore examined whether expression of the gene in both normal and tumour tissue was allelically balanced.

### AURKB AEI

**Figure 3:**
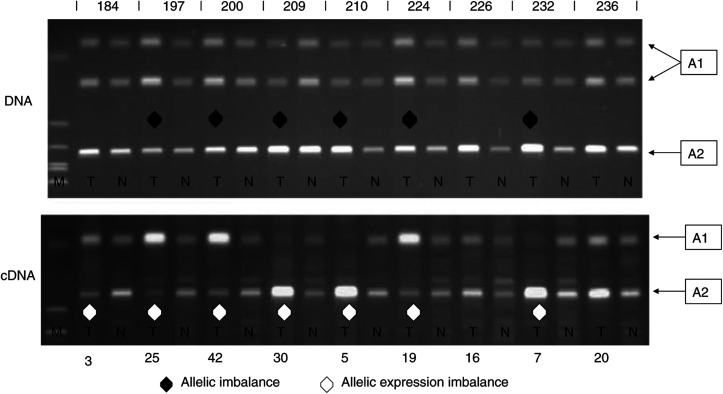
AURKB allele-specific expression analyses and corresponding allelic imbalance measurements in matched primary tissues from lung cancer patients. Most tumour samples showed expression strongly biased towards one allele. This is frequently accompanied (six out of seven) by a marginal allelic imbalance in the genomic DNA. The marker track (on all gel images) is a ØX174 *Hae*III digest. Bands representing parental alleles are marked A1/A2.

Nine patients (nine out of 44, 20%) were heterozygous for a nonsynonymous cSNP in exon 9 of the gene. Using PCR-RFLP and RT–PCR-RFLP analyses, we were able to show that relative to the patient matched normal cDNA, seven out of nine of these samples showed a marked AEI favouring one or other AURKB allele (outside the range of mean allele ratio in normal tissue±3s.d.). In six out of seven of these cases, this AEI was associated with a phase-matched allelic imbalance in the tumour DNA (Figure 3). The allelic ratio in the matched normal samples suggested that both alleles were genomically balanced and expressed equally in each patient.

### AURKB copy number analysis

Elevated gene expression for AURKA is associated in a number of tumours with high-level amplification of the gene (Sen *et al*, 1997; Sakakura *et al*, 2001). We therefore considered whether the high levels of expression of AURKB in lung cancer might also be related in some instances to high-level gene amplification. All tumour DNA samples were therefore scanned for such events by quantitative real time PCR. Using the MGMT locus on 10q26.3 as a genomic control, the mean Δ*C*_T_ (AURKB-MGMT) for 24 normal lung DNA samples was 0.4 with a range of 0.6 cycles. The mean Δ*C*_T_ for the 48 tumour DNA samples was significantly different at −0.3 (Student's *t*-test, *P*<3 × 10^−11^) and the range (2.2 cycles, maximally 4.5-fold copy number difference) was wider, suggesting that a number of the tumour samples had marginal increases in AURKB copy number. The Δ*C*_T_ of 17 out of 48 tumour DNA samples were outside the normal sample Δ*C*_T_ mean−3s.d. (indicating a small but significant relative over-representation of AURKB in these cases).

Taken together, these data suggest firstly that no tumour samples show high level amplification of the AURKB gene and, secondly, that a large number of tumours show apparent low level increases in gene copy number. Although we have no data suggesting that the MGMT locus undergoes homozygous deletion in NSCLC cells (an alternative possibility which would also explain our copy number data), we repeated the analysis on two samples showing apparently low levels of AURKB amplification using a second locus on a different chromosome as a control. Two samples (224, a tumour showing marginal allelic imbalance for AURKB, and 230) were analysed with their matched normal tissue and a formal ΔΔ*C*_T_ calculated against both control genes. The second genomic locus (HBG on 11p15.4) in this analysis also acted therefore as a control for MGMT stability. In both cases, the qPCR data suggested a low-level over-representation of the AURKB genomic locus in the tumour DNA relative to control levels :patient 224 : 1.9 and 1.7-fold and patient 230 : 2.9 and 2.5-fold (against MGMT and HGG1, respectively). These results therefore confirm that the MGMT locus is not affected by copy number differences in these tumours. The data also thereby confirm an increase in AURKB copy number has occurred in these tumours and further suggest that the allelic imbalance in case 224 was a consequence of additional copies of one allele and not deletion of the other in a subpopulation of tumour cells.

### Association of genetic instability with AURKB expression

*In vitro* data have indicated that the forced expression of aurora B kinase in Chinese hamster embryo cells is associated with increased expression of histone H3 and subsequent anueploidy (Ota *et al*, 2002). We therefore tested whether tumours with high levels of AURKB expression showed higher levels of genomic instability than those expressing low levels of the gene. Tumours were ranked initially according to their expression of AURKB as determined by comparative multiplex RT–PCR. The 16 highest and lowest expressing lesions were selected for analysis. One tumour DNA in each group was resistant to PCR amplification under the protocol used and so was excluded. A 10 marker microsatellite panel comprising loci previously shown to be highly informative and frequently imbalanced in lung carcinomas was used in multiplex analyses to amplify tumour and normal DNA from the 30 patients. Allelic imbalance for each marker, for each tumour, was scored according to previously defined criteria. The number of informative cases in each group showing either allelic imbalance or no allelic imbalance was totaled and the values compared between the two groups using a Fisher's exact test (Table 2). Tumours showing high levels of expression showed a highly significant excess of allelic imbalance (67 loci imbalanced *vs* 54 balanced) over those with low-level (42 imbalanced *vs* 81 balanced) expression (*P*=0.0012). To confirm this conclusion, these 30 cases were reranked using the Taqman qRT–PCR data (Table 1). While data were broadly similar, the real-time rankings resulted in the reciprocal movement of two cases between groups (194 and 231). Following this adjustment, the difference in overall allelic imbalance between the two groups was more marked (71 *vs* 50, 38 *vs* 85, respectively, *P*=0.0001).

Our earlier microarray analysis had indicated that AURKA was also frequently over-represented in primary NSCLC. Mindful of the suggestion of Keen and Taylor (2004) that AURKA and AURKB were generally coordinately expressed in human tumours, we analysed the expression levels of AURKA in the 30 patient series analysed for genetic instability by real time quantitative PCR. Although we were broadly able to affirm the conclusion of coordinate expression, there were clearly a number of tumours that expressed strongly one or other gene (Table 1). The 30 cases analysed for genetic instability were reranked according to AURKA expression and divided into two groups of low and high expression. The association of allelic imbalance observed with AURKB expression was no longer present in these AURKA-ranked groups (Table 2: 57 *vs* 66, 52 *vs* 69, *P*=0.8).

### AEI in other tumour-associated genes

**Figure 4:**
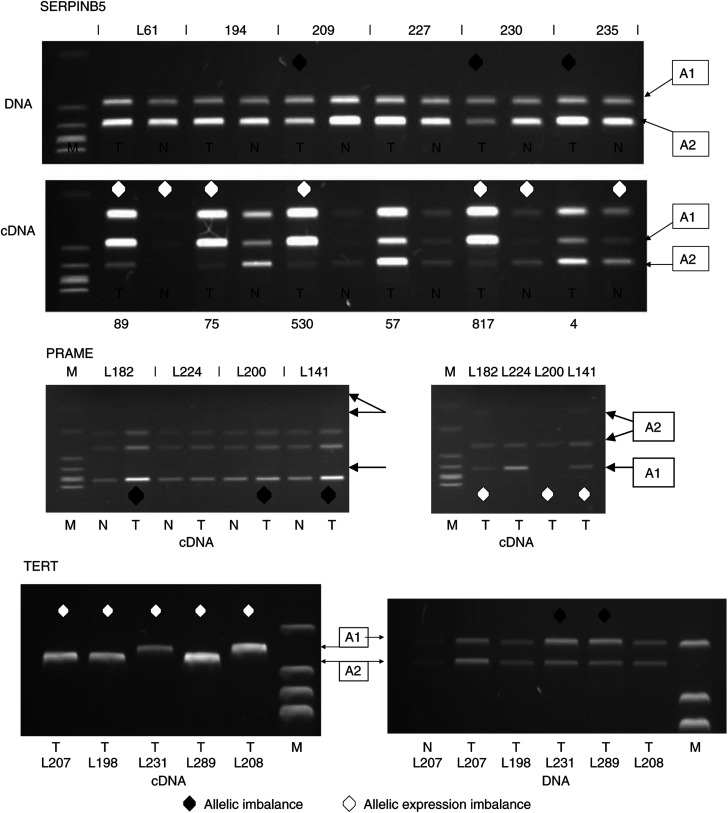
SERPINB5, PRAME and TERT allele-specific expression analyses and corresponding allelic imbalance measurements in matched primary tissues from lung cancer patients. SERPINB5: Two examples of tumours showing marked AEI in the apparent absence of AI are shown (L61, 194). In the case of L61, weak expression was seen in the matched normal tissue and this shows matching AEI. Patient 230 shows a phase matched AI and AEI in tumour tissue, a balanced normal DNA sample, and an AEI favouring the other allele in the normal cDNA. All samples from patient 227 are balanced. Real time values for over-representation of SERPINB5, relative to APPBP2 in the tumour over matched normal tissue are given below the image. PRAME: Three tumours showing AEI are shown with only L224 having equal co-expression of both alleles. TERT: Telomerase subunit expression was not detected in any normal lung tissue. 5 out of 5 informative samples analysed in duplicate runs showed expression strongly biased towards one parental allele.

Our previous analyses of the expression of two genes that are strongly expressed in tumours have suggested that these sequences are normally expressed in multipotent components of the bronchial airway (Smith *et al*, 2003, 2004). Given our observation of the overexpression of aurora B kinase and the suggestion that such expression might cause aneuploidy through chromosome miss-segregation, we analysed the allelic expression balance of a number of NSCLC associated sequences. The sequences chosen were generally amplifiable by RT–PCR only from tumour tissue, although we have shown that SERPINB5 is expressed in the normal airway basal cell (Smith *et al*, 2003). Both AEI and AI were measured in a series of informative patients using mRNA-encoded cSNPs for SERPINB5 (encoding maspin), TERT (encoding a telomerase subunit) and PRAME (encoding a tumour antigen of unknown function with a highly restricted normal tissue expression pattern (Heighway *et al*, 2002). In each case, we were able to identify high levels of AEI (Figure 4). For SERPINB5, AEI was seen in 15 out of 25 (60%) of informative tumours, 11 of which showed a phase-matched allelic imbalance. Four tumours in the series therefore demonstrated stronger expression from one parental allele in the apparent absence of AI. In a small number of cases only (5), it was possible to amplify maspin from matched histologically normal bronchial tissue. These cases may therefore represent an abnormal situation. In three of these samples, the cDNA showed AEI in the absence of phase-matched AI. In one case (230), tumour and normal tissue from the same patient showed phase miss-matched AEI. Allelic expression imbalance was also common (16 out of 18 samples) in tumours that expressed PRAME. Marginal (distinctive in one) AI was detected in nine out of 16 of those samples and in one cases, this marginal imbalance was phase miss-matched with the expression imbalance. Most dramatically, in the five out of five informative tumours where we were able to amplify the TERT cSNP, expression appeared to be overwhelmingly from one parental allele. In two cases this was associated with a clear allelic imbalance, which was phase miss-matched in one case.

## DISCUSSION

We have previously investigated the expression of a large number of transcripts in primary tumour *vs* normal tissue by microarray analysis (Heighway *et al*, 2002). Subsequently, by immunohistochemistry, we and others have begun to examine in normal lung tissue, preneoplastic lesions and primary tumours, the expression patterns of genes that are strongly, and apparently (as determined by RT–PCR) specifically, tumour expressed (Massion *et al*, 2003; Smith *et al*, 2003, 2004). The derived data show that many NSCLCs strongly express particular genes, including certain cytokeratins, which are characteristically expressed by the bronchial epithelial basal cells (Smith *et al*, 2004). In turn, such observations suggest that NSCLCs frequently derive from, share a common gene expression pattern with or tend to differentiate towards the basal cells. This cell type is a multipotent progenitor capable of renewing the mature epithelial cells of the airway (Hong *et al*, 2004).

A central question when examining such gene expression data, especially when as in most cases the expression pattern or even the identity of the cell of origin of the tumour is not known, is whether strong differential tumour expression in bulk tumour *vs* normal analyses simply reflects the typical expression pattern of the (rare) progenitor. In terms of the utility of a particular protein as a diagnostic or drug target, the reason why a candidate gene is strongly expressed in the tumour cell may be of secondary importance compared to how specifically that gene is expressed by the target cell and how vital that product is to host cell function. However, if our aim is to identify genes that are causally driving aspects of the neoplastic phenotype, then we need to identify those sequences which are expressed inappropriately as a consequence of induced DNA damage or erroneous epigenetic marking. Such information may increase our understanding of the disease process, guide future drug discovery or provide a rationale for the use of particular drugs in particular diseases.

Following such arguments, this study was an attempt to examine whether deregulation of aurora kinase genes, in particular AURKB, was implicated in lung cancer development. We have shown that AURKB is strongly expressed in exponentially proliferating bronchial epithelial cells in culture and that this expression is markedly reduced in confluent cells. We have also shown that almost all tumours show higher levels of AURKB (and AURKA) expression than their matched normal lung tissues, which could therefore simply be a consequence of a higher proliferative index, or be typical of the progenitor cell and atypical of the bulk of normal lung cells. But crucially, we have provided evidence that this expression is likely to be inappropriate given that in tumours but not matched normal tissue, it is generally strongly biased towards one parental allele. In many cases, this was associated with a marginal allelic imbalance at the genomic locus and a marginal increase in certain samples in locus copy number. As the *in vitro* bronchial cell preparation used in our analysis was uninformative for the cSNP, we could not confirm that the high level of gene expression seen in actively growing normal cells was bi-allelic. However, this conclusion is suggested by the analysis of AURKB expression in normal lung tissue from informative patients, where both alleles were expressed equally in each case. There are a number of reasons why the high-level expression of a gene in a tumour might occur predominantly from one of two genomically balanced alleles. These include *cis*-located mutational or epimutational differences, chromosomal translocation and polymorphism in tumour-utilised promoters or transcription factor binding sites. Further investigations are required to determine the cause(s) of the AEIs seen in AURKB and other genes implicated in NSCLC.

Further to the allelic expression data, we were able to show that patients with tumours expressing AURKB at the highest levels had a shorter progression-free survival. This observation supports the hypothesis that excessive AURKB expression is a marker for aggressive tumour biology. However, it must be stressed that this patient series is small and therefore this hypothesis must be viewed with some caution. Additional studies on larger patient series will be needed to more fully explore the role of AURKB expression as a potential prognostic marker for NSCLC patients.

The aurora family (A, B and C) are serine threonine kinases. Considerable attention is currently being given to the question of whether these proteins might be effective anticancer drug targets (for a review, Keen and Taylor, 2004). Genetic instability is a common feature of solid tumours. The molecular changes that lead to instability are imprecisely known, but are likely to involve defects in the process of mitosis. The protein kinases of the aurora family play a role in the regulation of mitosis from G2 through to cytokinesis and are implicated in the maintenance of correct chromosome number and accurate chromosome segregation. Alterations to the balance of expression of these genes could therefore have a role in cancer development through the generation of chromosome instability.

Aurora A kinase binds the centrosome and mitotic spindle and is involved in spindle assembly. Elevated expression has been documented in breast, bladder, colon, ovarian and pancreatic cancers (Zhou *et al*, 1998; Tanaka *et al*, 1999; Sen *et al*, 2002; Li *et al*, 2003) and gliomas (Klein *et al*, 2004). It has further been reported to correlate with invasive disease in breast cancer (Tanaka *et al*, 1999) and genomic instability in breast cancer (Miyoshi *et al*, 2001) and bladder cancer (Fraizer *et al*, 2004). The gene is amplified in many cancers including breast, bladder, heaptocellular and colorectal cancers and glioma and it has recently been implicated as a cancer-susceptibility gene in mouse and humans (Ewart-Toland *et al*, 2003). Forced expression of aurora A kinase in NIH3T3 cells and diploid human breast epithelial cells induced centrosome amplification and transformation *in vitro* and aurora A-transfected NIH3T3 cells formed tumours when injected into nude mice (Zhou *et al*, 1998).

Aurora B kinase is a chromosomal passenger protein that regulates chromosome segregation and cytokinesis. Its overexpression has been reported in several human cancer cell lines and in primary tumours including colorectal cancer (Bischoff *et al*, 1998), seminomas (Chieffi *et al*, 2004) and thyroid anaplastic carcinoma (Sorrentino *et al*, 2005). Exogenous expression of wild-type aurora B kinase in several tumour cell lines and in normal diploid fibroblasts has been shown to induce multinuclearity and aneuploidy (Tatsuka *et al*, 1998). Expression of kinase defective aurora B has similarly produced multinuclear cells resulting in cell-death (Terada *et al*, 1998).

The function of aurora C kinase is less well defined. The gene is overexpressed in primary colorectal cancer and a variety of cell lines (Kimura *et al*, 1999; Takahashi *et al*, 2000).

Anueploidy is one of the defining characteristics of malignant cells. As aurora B kinase appears to have a critical role in ensuring the accurate segregation of chromosomes at mitosis through the destabilisation of syntelic (not under tension) attachments (Dewar *et al*, 2004) and as Ota *et al* (2002) had shown in a model system that forced expression the protein was linked to the generation of aneuploidy, we examined whether tumours with high levels of AURKB expression might show higher levels of genomic instability. While our analysis was limited, this did indeed appear to be the case. This raises an intriguing question: could early deregulation of AURKB, resulting in either inappropriately high levels of or else inappropriately timed expression of this protein be a driver of chromosome instability in tumours? This is an attractive hypothesis although the alternative explanation, that tumours with higher levels of genetic instability might be those which tend to show low level amplification (generating allelic imbalance and perhaps AEI) and high levels of expression of AURKB, cannot be discounted without further analysis. If deregulation of AURKB expression is indeed an early event promoting genetic instability, then anti aurora B kinase agents may have application in the future not only in the treatment of advanced invasive disease but also in the suppression of the development of disease in high-risk patients (secondary chemoprevention).

As we have noted already, a number of microarray highlighted tumour expressed genes are normally expressed by multipotent components of the airway but not by their differentiated progeny. For a number of these tumour-expressed genes, we have also shown that at high frequency, expression in tumour tissue is strongly biased towards one chromosomal allele. Taking SERPINB5 (encoding maspin) as a specific example, we reasoned that the rare expression of such genes in histologically normal differentiated cells and more commonly in preneoplastic lesions (Smith *et al*, 2003) and tumours might be a consequence of a failure to appropriately inactivate these sequences in differentiation-committed progeny during the asymmetric division of multi-potent cells (Heighway *et al*, 2004). SERPINB5 expression in normal tissue is known to be controlled at least in part by promoter methylation (Futscher *et al*, 2002). The common failure to inactivate one or both alleles of a gene that is associated with maintenance of stem cell multi-potentiality in a differentiating cell (an event perhaps linked causally to preneoplasia) and maintenance of that imprint through tumorigenesis might explain our data. However, given our observations concerning AURKB deregulation in tumours and the association of high levels of expression with genetic instability in those lesions, we must consider the possibility that the segregation of epigenetically marked chromatin is error prone in multipotent tumour progenitors and that the deregulation of AURKB contributes to that process.

Elegant experimental evidence exists that a stem cell chromatin strand is retained in the stem cell progeny of asymmetric divisions (Cairns, 1975; Merok *et al*, 2002; Potten *et al*, 2002). It is therefore likely that this strand retains the epigenetic marking associated with the multipotent cell gene expression pattern. Correspondingly, it would seem likely that a new epigenetic imprint appropriate for differentiation-committed cell gene expression is applied to the non-stem cell chromatin. If chromosomes were miss-segregated to the wrong daughter during asymmetric division, perhaps as a consequence of miss-regulated expression of aurora B kinase, this might therefore lead to a blurring of gene expression patterns with perhaps the retention of certain stem cell characteristics by differentiation-committed daughter cells. Such miss-segregation could conceivably be followed by chromosomal recombination events that would generate a chromatin mosaic. This hypothesis, which might explain many of the gene expression and epigenetic chromatin modification patterns of malignant cells, clearly requires extensive further testing.

We believe that this work represents the first comprehensive analysis of the expression of AURKB in primary NSCLC. We further believe that the study provides some measure of rational justification for the testing of aurora B kinase inhibitors in the treatment of invasive lung cancer.

## Figures and Tables

**Table 1:** *Expression data and sample details:* Patients are ranked according to the comparative multiplex RT-PCR values for AURKB (column 2)

	**Aurora B**		**Aurora A**			
**Patient**	**cmRT-PCR**	**Real time**	**Real time**	**Gender**	**Age**	**Tumour type**
195	0.3	0.4	37	M	65.4	Squamous cell
179	0.8	1	0.9	M	47.8	Squamous cell
187	1	1		F	51.8	Adeno
206	2	1	0.6	M	59.5	Squamous cell
210	2	5	23	M	52.6	Adeno
223	2	5	6	M	67.2	Adeno
173	3	2	50	M	73.4	Adeno
182	4	4	9	M	69.5	Squamous cell
176	4	5	2	F	57.4	Adeno
231	6	17	0.07	M	72.5	NSCLC
232	6	7	0.5	M	74.3	Adeno
184	6	3	8	F	39.2	Adeno
211	6.8	1	2	M	71.5	Adeno
208	7	4	6	M	47.6	Squamous cell
205	8	11	3	M	73.3	Squamous cell
216	8	10	6	M	57.7	Squamous cell
						
217	9	11		F	69.5	Adeno
237	12	12		M	79.5	Squamous cell
224	13	19		M	68.5	Squamous cell
222	13	21		M	54.6	Adeno
226	14	16		F	62.6	Adeno
186	15	14		M	68.5	Adeno
227	16	17		M	74.8	Squamous cell
214	16	39		F	69.7	Adeno
236	17	20		F	80.6	Adeno
189	17	17		F	57.1	Adeno
233	18	18		F	50.4	Adeno
218	19	18		M	72	Adeno
						
235	27	42	0.001	M	65.6	Large cell
198	28	29	65	M	74.6	Squamous cell
194	29	14	14	M	68.9	Squamous cell
229	29	16	0.1	M	80.7	Adeno
193	30	21	7	M	74.8	Squamous cell
200	32	42	34	M	62.6	Adeno
207	36	721	2589	M	63.3	Squamous cell
183	40	53	25	M	63.7	Adeno
174	42	65	40	M	49.6	LCNEC
209	44	30	125	M	55.7	Squamous cell
204	47	81	22	M	58	Squamous cell
197	64	25	14	M	54.7	Squamous cell
230	76	108	40	M	70.1	Squamous cell
220	80	105	12	M	76.2	Squamous cell
175	144	85	—	F	65.2	Adeno
196	189	53	7	M	52.9	Adeno

The corresponding real time values are listed in column 3 and the AURKA real time data for the two groups analysed for genetic stability are given in column 4. Values are fold-change in the tumour relative to patient matched normal tissue, normalised by APPBP2 levels.

**Table 2:** *Microsatellite derived instability data:* loci scored as allelically imbalanced were totalled in each group and compared

The top panels represent low and high relative expression of AURKB as determined by comparative multiplex RT–PCR. The lower panel represents those cases re-ranked into similar groups on the basis of AURKA expression.
